# Supplementary material for: Patterns of Intron Gain and Loss in Fungi
Source: PLoS Biol. 2004 Nov 30;2(12):e422. doi: 10.1371/journal.pbio.0020422 (PMC532390; doi:10.1371/journal.pbio.0020422)
Supplement: Table S1 — Also available at http://genes.mit.edu/NielsenEtAl/. (4.3 MB ZIP). [file pbio.0020422.st001.zip › NielsenEtAl/html/351.html]

AN6650.1.NCU02482.1.MG02617.1.FG00175.1


```
 CLUSTAL W (1.82) Multiple Sequence Alignments - Introns Inserted


Sequence 1: MG02617.1	458 aa
Sequence 2: FG00175.1	472 aa
Sequence 3: NCU02482.1	470 aa
Sequence 4: AN6650.1	460 aa
Alignment Length: 473 aa
Number Identitical Residues: 295 aa
Alignment Score (without introns) 13717


MG02617.1 	--MP1CTSNVRSTRNYS~-~------------ASSEPDLKTTLKSVIPAKRELLKKVKAH
NCU02482.1	MAMN~LRTGSRAFGGAL~R0N--HARVSRRGYATAEPDLKTVLKEAIPEKRELFKKVKAH
FG00175.1 	MALN~LTTSRRALSSLK0P~LTRAAFVGARGYATAEPDLKATLREAIPAKRELLKKVKAH
AN6650.1  	MALP~LRTARHASRLAQ0T~IG------RRGYATAEPDLKSALKAVIPAKRELLAEVKKQ
          	 ::    :  ::                  . *::*****:.*: .** ****: :** :

MG02617.1 	GSKVIGEVKVENTIGGMRGLKAMVWEGSVLDADEGIRFHGRTIKDCQRELPKGKTGTEML
NCU02482.1	GNKVLGEVKVENTIGGMRGLKAMIWEGSVLDANEGIRFHGRTIKDCQKELPKGKNGTEML
FG00175.1 	SNKVLGEVKVENTLGGMRGLKAMVWEGSVLDANEGIRFHGRTIKDCQKELPKGKTGTEML
AN6650.1  	GDEVIGEVKVSNVIGGMRGLKSMLWEGSVLDADEGIRFHGKTIKDCQKELPKGPTGTEML
          	..:*:*****.*.:*******:*:********:*******:******:***** .*****

MG02617.1 	PEAMFWLLLTGQVPSTNQVRVLSRQLAEQAQLPAFVNKMLDDFPRDLHPMTQFAIAVSAL
NCU02482.1	PEAMFWLLLTGKVPSTNQVRQFSRELAEQAALPDFVNKLLDGFPKDLHPMTQFAIAVSAL
FG00175.1 	PEAMFWLLLTGQVPSVNQVRGFSRELAEKAQIPEFVSKMLNDFPKDLHPMTQFAMAVSAL
AN6650.1  	PEAMFWLLLTGEVPSTSQVRAFSKQLAEESHLPDHILDLAKSFPKHMHPMTQISIITAAL
          	***********:***..*** :*::***:: :* .: .: ..**:.:*****::: .:**

MG02617.1 	NHTSKFAKAYADGLNKADYWEPTFDDSISLLAKLPTIAAKIYHNSYKGGGALPAEVDLEQ
NCU02482.1	NHTSKFAKAYEQGLNKADYWEPTFDDCISLLGKLPTIAAKIYQNSYKGGGALPAEVDLEQ
FG00175.1 	NYESKFAKAYEQGINKADYWEPTFDDCISLLAKLPTIAAKIYQNAYRGGGALPAEVDLEQ
AN6650.1  	NTESKFAKLYEKGINKADYWEPTFDDAISLLAKIPRVAALVFR-PNEIDVVGRQKLDPAQ
          	*  ***** * .*:************.****.*:* :** ::: . . . .   ::*  *

MG02617.1 	DWSYNFAAMLGKGGKGNENFQDLLRLYLALHGDHEGGNVSAHATHLVGSALSDPFLSYSA
NCU02482.1	DWAYNFAAMLGKPGKENENFQDLLRLYLALHGDHEGGNVSAHTTHLVGSALSDPFLAYSA
FG00175.1 	DWSYNFAAMLGKGGKENENFQDLLRLYLALHGDHEGGNVSAHATHLVGSALSDPFLSYSA
AN6650.1  	DWSYNFAELLGKGGANNADFHDLLRLYLALHGDHEGGNVSAHATHLVGSALSDPFLSYSA
          	**:**** :*** *  * :*:*********************:*************:***

MG02617.1 	GLQGLAGPLHG2LAAQEVLRWIIQMKEAIPATYSEKDVTDYLWSTLNSGRVVPGYGHAVL
NCU02482.1	GLQGLAGPLHG~LAAQEVLRWILQMKEAIPSSYTDEDVRSYLWNTLNSGRVVPGYGHAVL
FG00175.1 	GLQGLAGPLHG2LAAQEVLRWIIQMKEAIPSNYTEQDVNDYLWSTLNSGRVVPGYGHAVL
AN6650.1  	GLLGLAGPLHG~LAAQEVLRWILAMQEKIGTKFTDEDVRAYLWDTLKSGRVVPGYGHGVL
          	** ******** **********: *:* * :.::::**  ***.**:**********.**

MG02617.1 	RKPDPRFQALMDYAASRPEIAKDPVFQLVQLNSQVAPEVLKKHGK~TKNPYPNVDSSSGV
NCU02482.1	RKPDPRFEALMDYAASRPKIAADPVFQLVLKNSQIAPEVLKQHGK~TKNPYPNVDSSSGV
FG00175.1 	RKPDPRFEALMDYAAARPEIANDPVFQLVEKNSRIAPEVLKKHGK~TKNPYPNVDSSSGV
AN6650.1  	RKPDPRFQALMDFAATRKDVLANPVFQLVKKNSEIAPGVLTEHGK0TKNPHPNVDAASGV
          	*******:****:**:* .:  :******  **.:** **.:*** ****:****::***

MG02617.1 	LFHHYGFHETLYYTATFGVSRGLGPLAQLIWDRALGLPIERPKSINLEGILKIVGDKS
NCU02482.1	LFHHYGFHETLYYTATFGVSRGLGPLAQLIWDRALGLPIERPKSINLEGILKQVEGQ-
FG00175.1 	LFHHYGFHETLYYTATFGVSRGLGPLAQLIWDRALGLPIERPKSINLEGILKQVEGQ-
AN6650.1  	LFYHYGFQQPLYYTVTFGVSRALGPLVQLIWDRALGLPIERPKSINLLGLKK------
          	**:****::.****.******.****.******************** *: *
```
